# Iron overload impairs renal function and is associated with vascular calcification in rat aorta

**DOI:** 10.1007/s10534-022-00449-7

**Published:** 2022-09-30

**Authors:** Yanqiu Song, Ning Yang, Hailong Si, Ting Liu, Hongyu Wang, Hua Geng, Qin Qin, Zhigang Guo

**Affiliations:** 1 Cardiovascular Institute, Tianjin Chest Hospital, Tianjin, 300222 China; 2 Department of Cardiology, Tianjin Chest Hospital, Tianjin, 300222 China; 3 Department of Cardiovascular Surgery, Tianjin Chest Hospital, Tianjin, 300222 China; 4 Department of Pathology, Tianjin Chest Hospital, Tianjin, 300222 China

**Keywords:** Iron overload, Vascular calcification, DMT1, FPN1, Hepcidin, Aorta

## Abstract

Vascular calcification (VC) has been associated with a risk of cardiovascular diseases. Iron may play a critical role in progressive VC. Therefore, we investigated the effects of iron overload on the aorta of rats. A rat model of iron overload was established by intraperitoneal injection of Iron-Dextran. The levels of iron, calcium, and ALP activity were detected. Von Kossa staining and Perl’s staining were conducted. The expression of iron metabolism-related and calcification related factors were examined in the aortic tissue of rats. The results showed serum and aortic tissue iron were increased induced by iron overload and excessive iron induced hepatic and renal damage. In iron overload rats, the expression of divalent metal transporter 1 (DMT1) and hepcidin were higher, but ferroportin1 (FPN1) was lower. Von Kossa staining demonstrated calcium deposition in the aorta of iron overload rats. The calcium content and ALP activity in serum and aortic tissue were increased and iron level in aortic tissue highly correlated with calcium content and ALP activity. The expressions of the osteogenic markers were increased while a decrease of Alpha-smooth muscle actin (α-SMA) in the aortic tissue of iron overload rats. IL-24 was increased during the calcification process induced by iron. Overall, we demonstrated excessive iron accumulation in the aortic tissue and induced organs damage. The iron metabolism-related factors were significantly changed during iron overload. Moreover, we found that iron overload leads to calcium deposition in aorta, playing a key role in the pathological process of VC by mediating osteoblast differentiation factors.

## Introduction

Iron is an essential trace element that participates in the synthesis of various functional proteins, playing an important role in the normal physiological and metabolic activities of our bodies (Muckenthaler et al. 2017). It has been established that iron homeostasis is tightly regulated, and both iron deficiency and iron overload can threaten human health and even cause serious diseases (Dev and Babitt 2017; Fleming and Ponka 2012).

Vascular calcification (VC) is widely acknowledged as a common pathological manifestation of atherosclerosis, hypertension, diabetic vascular disease, vascular damage, chronic kidney disease and aging (Leopold 2015). Importantly, coronary artery calcification (CAC) volume has been reported to be positively and independently associated with cardiovascular disease risk in the Multi-Ethnic Study of Atherosclerosis (MESA) (Criqui et al. 2014). Nonetheless, the mechanisms underlying VC remain unclear, warranting further studies.

Robust evidence suggests that iron accumulates in the aortic valve and actively contributes to aortic valve calcification (Andres et al. 2016; Morvan et al. 2019), indicating that iron may play a critical role in progressive VC. However, conflicting results have been reported from studies that examined the role of iron in calcification in vascular smooth muscle cells (VSMCs) models. It has been shown that calcification of human aortic smooth muscle cells is synergistically enhanced by iron and TNF-alpha stimulation (Kawada et al. 2018). However, iron citrate reportedly inhibits VSMCs calcification induced by high phosphate via prevention of apoptosis and autophagy (Ciceri et al. 2016, 2019). Wang et al. reported that iron sucrose alleviates VSMCs calcification induced by high phosphate media in a dose-dependent manner and leads to iron overload in the vasculature at high concentrations (Wang et al. 2021).

Overall, the role of iron on VC remains largely unclear. To the best of our knowledge, few studies have focused on the role of iron overload on VC in vivo. In this study, we assessed the expression of iron mediators in the aorta of iron overload rat models and the potential role of iron overload on rat aorta.

## Materials and methods

### Animals

Twenty 8-week-old male Sprague Dawley rats weighing 200–250 g each were purchased from BEIJING HFK BIOSCIENCE Co., Ltd. (license number: SCXK (Beijing) 2019–0008). The rats were housed for 1 week under the following conditions: 18–25 °C; 40–60% humidity; 12:12 light/dark cycle; ad libitum diet comprising standard laboratory rat feed (about 0.6–1.2% phosphorus and 1.0–1.8% calcium) and water. Rats were randomly divided into two groups. Rats in the iron overload group (n = 10) received an intraperitoneal injection of Iron-Dextran (250 mg/Kg of body weight, Sigma) 5 days a week for 4 weeks to establish a rat model of iron overload. In contrast, rats in the control group (n = 10) received an intraperitoneal injection of the same dose of 0.9% NaCl solution. The animal experiments were approved by the Animal Experiments Committee of Tianjin Chest Hospital (Approval Number: TCH-2021-003).

### Serum biochemistry parameters testing

The blood urea nitrogen (BUN), creatinine (CRE), aspartate transaminase (ALT), aspartate transaminase (AST), total protein (TP), albumin (Alb), glucose (Glu), C-reactive protein (CRP), total cholesterol (TC), triglyceride (TG), high density lipoprotein (HDL), low density lipoprotein (LDL), very low density lipoprotein (VLDL) and inorganic phosphorus (iP) were examined with an automatic biochemical analyzer (Roche Cobas c701, Basel, Switzerland). The serum levels of intact parathormone (I-PTH) was determined using ELISA kits (E-EL-R0535c, Elabscience Biotechnology Co., Ltd, Hubei, China) according to the manufacturer’s instructions.

### Alkaline phosphatase (ALP) activity, iron concentration and calcium content assay

The serum levels of iron, calcium and ALP activity were detected in the present study. 2 mL of venous blood was collected in a coagulation tube. Serum was isolated by centrifugation at 4 °C and 1810×*g* for 10 min, and stored in a metal-free tube at − 80 °C. The Alkaline phosphatase assay kit (A059-2), serum iron assay kit (A039-1-1) and calcium assay kit (C004-2-1) were purchased from Nanjing Jiancheng Bioengineering Institute (Jiangsu, China). The serum levels of iron, calcium and ALP activity were determined according to the manufacturer’s instructions.

The iron, calcium, and ALP activity levels were also detected in aortic tissue. Approximately 100 mg of aortic tissue was homogenized in pre-cooled physiological saline; the supernatant was then isolated by centrifugation at 4 °C and 1257×*g* for 10 min. The Alkaline phosphatase assay kit (A059-2), tissue iron assay kit (A039-2-1) and calcium assay kit (C004-2-1) were purchased from Nanjing Jiancheng Bioengineering Institute (Jiangsu, China). The ALP activity, iron concentration and calcium content in aortic tissue were determined according to the manufacturer’s instructions. The results were normalized by the protein content determined by the BCA protein assay kit (P0012s, Beyotime Biotechnology, Shanghai, China).

### Von Kossa staining

For Von Kossa staining, the rat aortic rings were cut into 5 μm-thick paraffin-embedded sections. The dewaxed sections were stained with Von Kossa silver solution (DS0003, Beijing Leagene Biotechnology Co., Ltd, Beijing, China) under an ultraviolet lamp for 20 min. Then dewaxed sections were stained with hematoxylin for 3 min, washed with water for 2 min, differentiated in mild acid for 30 s, and washed with water again. After staining with 1% ammonia for 3 min, the tissue sections were washed and stained with eosin for 45 s. After dehydration, the tissue sections were placed under a cover slip. Pathological changes were observed using an optical microscope (Olympus Corporation, Tokyo, Japan). The calcium deposit area was stained in black or brown-black, the nucleus was blue, and the background was red.

### Perl’s staining

For Perl’s staining, the rat aortic rings were cut into 5 μm-thick paraffin-embedded sections. The dewaxed sections were stained with Perl’s stain (DJ0002, Beijing Leagene Biotechnology Co., Ltd, Beijing, China) for 20 min. Then the dewaxed sections were stained with eosin for 30 s. Pathological changes were investigated using an optical microscope (Olympus Corporation, Tokyo, Japan). Ferric iron was stained blue, and cell nuclei and other tissues were red.

### Immunofluorescence (IF) assay

Rat aorta tissue samples were fixed in 4% paraformaldehyde for 24–48 h, embedded in paraffin, and mounted on slides (4 µm-thick coronal sections). The tissue slices were dewaxed twice in xylene for 10 min and dehydrated in a gradient ethanol solution (100, 95, 80, and 70%). Slices were rinsed with PBS 3 times for 2 min each. Antigen retrieval was performed in citrate antigen retrieval solution (C1032, Solarbio Life Sciences, Beijing, China) at 95 °C for 10 min in a pressure cooker. After allowing the slices to cool naturally in the retrieval solution, they were rinsed with PBS, followed by blocking with 5% bovine serum albumin at room temperature for 2 h, and the excess liquid was shaken off. The primary antibodies of target cells and cytokines were added at 4 °C overnight. The primary antibodies used consisted of rabbit anti-Ferroportin/SLC40A1 (1:500; NBP1-21502, Novus Biologicals, Colorado, USA), rabbit anti-DMT1 (1:300; bs-3577R, BEIJING BIOSYNTHESIS BIOTECHNOLOGY CO., LTD., Beijing, China), and mouse anti-alpha smooth muscle actin antibody (1:200; BM0002, Boster Biological Technology co. ltd, Hubei, China). The primary antibody was removed, and slices were rinsed with PBS 3 times for 3 min each. The indicated fluorescence-labeled secondary antibodies were added and incubated at room temperature for 1 h in the dark. The secondary antibodies used consisted of DyLight 488 conjugated AffiniPure donkey anti-rabbit IgG (H + L) (BA1145) and DyLight 488 conjugated AffiniPure donkey anti-mouse IgG (H + L) (BA1146, Boster Biological Technology co. ltd, Hubei, China). Finally, slices were mounted with an anti-fade mounting medium with 4,6-diamidino-2-phenylindole (S2110, Solarbio Life Sciences, Beijing, China) and photographed with a fluorescence microscope. Each section was placed under an Olympus inverted fluorescence microscope (BX51) for observation, and three images were selected randomly at × 200 magnification. Image pro-plus 6.0 software was used to analyze the integrated optical density (IOD) of each fluorescent image, and the average IOD was calculated.

### Quantitative reverse-transcription polymerase chain reaction (qRT-PCR) analysis

Total RNA was extracted from approximately 100 mg of aortic tissue with TRIzol Reagent (REF 15596026, Ambion, Life technologies Corporation, Carlsbad, USA) according to the manufacturer’s instructions. Two micrograms of RNA were reverse transcribed using a Revert Aid First Strand cDNA Synthesis Kit (#K1622, Thermo Fish Scientific Inc, Massachusetts, USA) according to the manufacturer’s instructions. Quantitative PCR was performed using an Applied Biosystems 7500 real-time PCR system with SYBR Premix Ex Taq II (RR820; TaKaRa Bio Inc., Kusatsu, Japan). The 2^−ΔΔCt^ method (Livak and Schmittgen 2001) was used to calculate the relative levels of target mRNA, and the gene that encodes glyceraldehyde-3-phosphate dehydrogenase (GAPDH) was used as an internal control. The PCR primers (AUGCT Biotechnology Co., Inc., Beijing, China) are listed in Table 1.

**Table 1:** Primer sequences used in the qRT-PCR analysis

Gene	Forward	Reverse
DMT1	5′-ACTCTTCCCTCCCACATTCCA-3′	5′-TCCAGGTAGGCAATGCTCATAAG-3′
FPN1	5′-GAAAACAGGAGCAGATTAGCAGACA-3′	5′-GAAATGAAACCACAGCCAATGAC-3′
Hepcidin	5′-GAAGGCAAGATGGCACTAAGCA-3′	5′-TCTCGTCTGTTGCCGGAGATAG-3′
ACTA2	5′-AGGGCTGTTTTCCCATCCAT-3′	5′-GCTGTCCTTTTGGCCCATT-3′
RUNX2	5′-CATGGCCGGGAATGATGAGA-3′	5′-GAAACTCTTGCCTCGTCCG-3′
BMP2	5′-AGAAAAGCGTCAAGCCAAACAC-3′	5′-CCCACATCACTGAAGTCCACATAC-3′
Msx-2	5′-AAGGCAAAAAGACTGCAGGA-3′	5′-GGATGGGAAGCACAGGTCTA-3′
RANKL	5′-TCGGGTTCCCATAAAGTCAG-3′	5′-CTGAAGCAAATGTTGGCGTA-3′
OPN	5′-AGTGGTTTGCTTTTGCCTGTTC-3′	5′- TCTGAGATGGGTCAGGCTTCA-3′
IL-24	5′-TGTTCTCCGTGCCATTTCAA-3′	5′-CATGGCTGTCGTTTGGACTAAA-3′
GAPDH	5′-ACAAAGTGGACATTGTTGCC-3′	5′-AAACATGGTGGTGAAGACGC-3′

### Western blot analysis

Approximately 100 mg of aortic tissue was homogenized in RIPA buffer (R0010, Solarbio Life Sciences, Beijing, China) with protease inhibitors and phosphatase inhibitors cocktail (Roche, Mannheim, Germany). The homogenates were centrifuged, and the protein concentration was determined using a BCA protein assay kit (P0012s, Beyotime Biotechnology, Shanghai, China). The protein samples (30 μg per well) were separated on 10% sodium dodecyl sulfate–polyacrylamide gel and transferred to polyvinylidene difluoride membranes (Merck Millipore Ltd., Darmstadt, Germany). The membranes were blocked with 5% non-fat milk for 2 h at 25 °C and incubated overnight with primary antibodies at 4 °C. The primary antibodies included rabbit anti-BMP-2 (1:500; bs-1012R, BEIJING BIOSYNTHESIS BIOTECHNOLOGY CO., LTD., Beijing, China), mouse anti-RUNX2 (1:500; sc-101145), mouse anti-OPN (1:500; sc-21742), mouse anti-Msx-2 (1:500; sc-365232), mouse anti-RANKL (1:500; sc-377079) (Santa Cruz Biotechnology, Dallas, Texas, USA), rabbit anti-Hepcidin (1:1000; ab30760) and mouse anti-glyceraldehyde-3-phosphate dehydrogenase (1:5000; ab8245) (Abcam, Discover Drive, Cambridge Biomedical Campus, Cambridge, UK). Subsequently, the membranes were incubated with secondary antibodies for 1 h at room temperature. The secondary antibodies included goat anti-rabbit IgG horseradish peroxidase (1:5000; ab205718) and goat anti-mouse IgG horseradish peroxidase (1:5000; ab205719) (Abcam, Discover Drive, Cambridge Biomedical Campus, Cambridge, UK). The membranes were incubated with Pierce™ ECL Western Blotting Substrate (Thermo, Thermo Scientific., Rockford, USA) for 1 min. The protein bands were visualized using a ChemiDOC™ XRS + System (Bio-Rad Laboratories Inc., California, USA) and quantified by densitometry.

### ELISA

The serum levels of Ferritin (F048SC) and Total Iron Binding Capacity (TIBC) (T181SC) were determined using ELISA kits (HCB, ELIXIR CANADA MEDICINE COMPANY LTD, Vancouver B.C, Canada) according to the manufacturer’s instructions. The optical density of each solution at 450 nm was determined using a Multiskan Mk3 Microplate Reader (Thermo, Massachusetts, USA). The concentration of each test sample was calculated from the standard curve. The percentage saturation of transferrin with iron (TSAT) was calculated by dividing the serum iron concentration by TIBC.

The IL-24 levels in aortic tissue was determined with ELISA kits (E2064r, EIAAB SCIENCE INC, Hubei, China) according to the manufacturer’s instructions.

### Statistical analysis

All data were statistically analyzed using SPSS 16.0 software and expressed as mean ± standard deviation. The Shapiro–Wilk test was used to assess if the data were normally distributed. Differences between the two groups were determined by the Student’s *t*-test. Correlational analyses were assessed by Pearson correlation coefficients (*r*^2^). A *P*-value < 0.05 was statistically significant.

## Results

### Iron levels in serum and aortic tissue

**Fig. 1:**
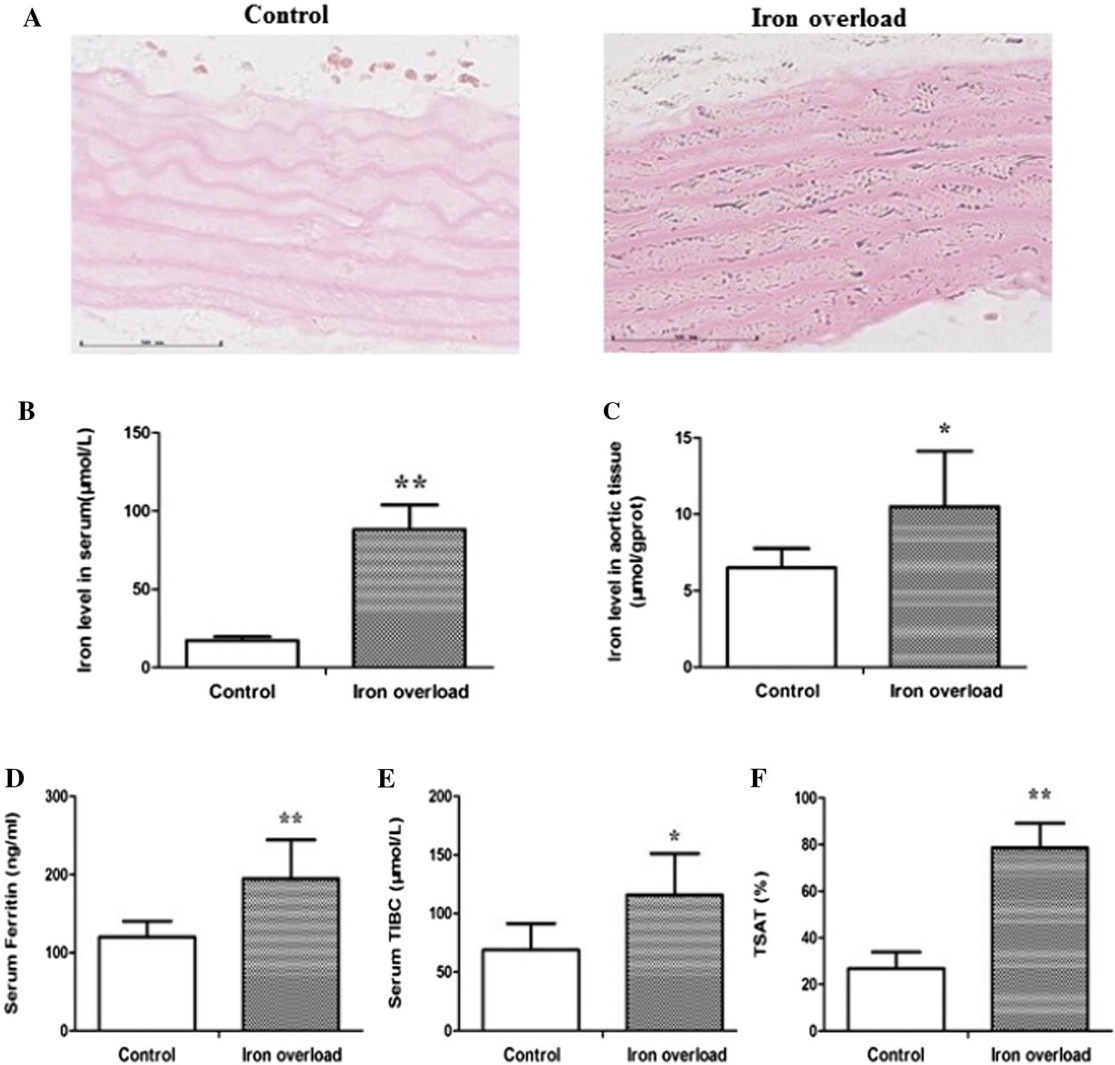
Iron levels in serum and aortic tissue. **A** Perl’s Staining of rats’ aorta (400 × magnification), **B** Iron levels in serum, **C** Iron levels in aortic tissue, **D** Serum Ferritin levels, **E** Serum TIBC, **F** TSAT. Data are expressed as means ± SD; n = 6 rats per group. *P < 0.05; **P < 0.01 *vs.* the control group

To determine the iron levels in rat models of iron overload, we assessed aortic tissue iron deposition. Perl’s staining demonstrated blue-stained iron deposits in aortic tissue in the rat models of iron overload (Fig. 1A). We then examined the iron levels in the serum and aortic tissue. We found that iron levels in serum (88.165 ± 15.830 μmol/L) and aortic tissue (10.494 ± 3.636 μmol/g protein) were higher in the iron overload group than in the control group (17.338 ± 2.289, 6.507 ± 1.259) (*P* < 0.05, Fig. 1B, C). The serum Ferritin (194.816 ± 49.572 ng/ml) and TIBC (115.959 ± 35.476 nmol/L) levels were also higher in the iron overload group than in the control group (120.017 ± 20.240, 69.127 ± 22.411) (*P* < 0.05, Fig. 1D, E). Moreover, significantly higher TSAT levels were found in the iron overload group (78.640 ± 10.564%) than in the control group (26.888 ± 7.076%) (*P* < 0.01, Fig. 1F).

### Serum biochemical parameters

There were no significant differences in TP, Glu, CRP, TC, TG, HDL, LDL, and VLDL between the control and iron overload group (Table 2). However, in iron overload group the serum BUN (*P* < 0.01), CRE (*P* < 0.05), ALT (*P* < 0.01), AST (*P* < 0.05), and iP (*P* < 0.01) were increased compared with the control group. The serum Alb and I-PTH were significantly lower induced by iron overload (*P* < 0.01).

**Table 2:** Serum biochemical markers

	Control	Iron overload
BUN (mmol/L)	4.200 ± 0.510	5.257 ± 0.526**
CRE (μmol/L)	18.500 ± 2.429	22.167 ± 2.784*
ALT (U/L)	33.686 ± 4.679	41.971 ± 4.340**
AST (U/L)	119.014 ± 52.946	200.014 ± 47.776*
TP (g/L)	54.220 ± 2.414	51.720 ± 1.045
Alb (g/L)	39.467 ± 2.157	35.383 ± 1.141**
Glu (mmol/L)	7.096 ± 2.419	7.491 ± 2.183
CRP (mg/L)	0.106 ± 0.014	0.113 ± 0.015
TC (mmol/L)	1.522 ± 0.184	1.720 ± 0.180
TG (mmol/L)	1.346 ± 0.290	1.284 ± 0.362
HDL (mmol/L)	0.892 ± 0.112	0.800 ± 0.087
LDL (mmol/L)	0.213 ± 0.076	0.257 ± 0.081
VLDL (mmol/L)	0.500 ± 0.096	0.552 ± 0.039
I-PTH (pg/mL)	413.112 ± 52.721	353.737 ± 21.360*
iP (mmol/L)	2.218 ± 0.383	4.977 ± 0. 935**

*BUN* Blood urea nitrogen, *CRE* Creatinine, *ALT* Aspartate transaminase, *AST* Aspartate transaminase, *TP* Total protein, *Alb* Albumin, *Glu* Glucose, *CRP* C-reactive protein, *TC* Total cholesterol, *TG* Triglyceride, *HDL* High density lipoprotein, *LDL* Low density lipoprotein, *VLDL* Very low density lipoprotein, *I-PTH* Intact parathormone, *iP* Inorganic phosphorus. Data are expressed as means ± SD; n = 6–7 rats per group. *P < 0.05; **P < 0.01 *vs.* the control group

### Expression of iron metabolism-related factors in aortic tissue of iron overload rats

**Fig. 2:**
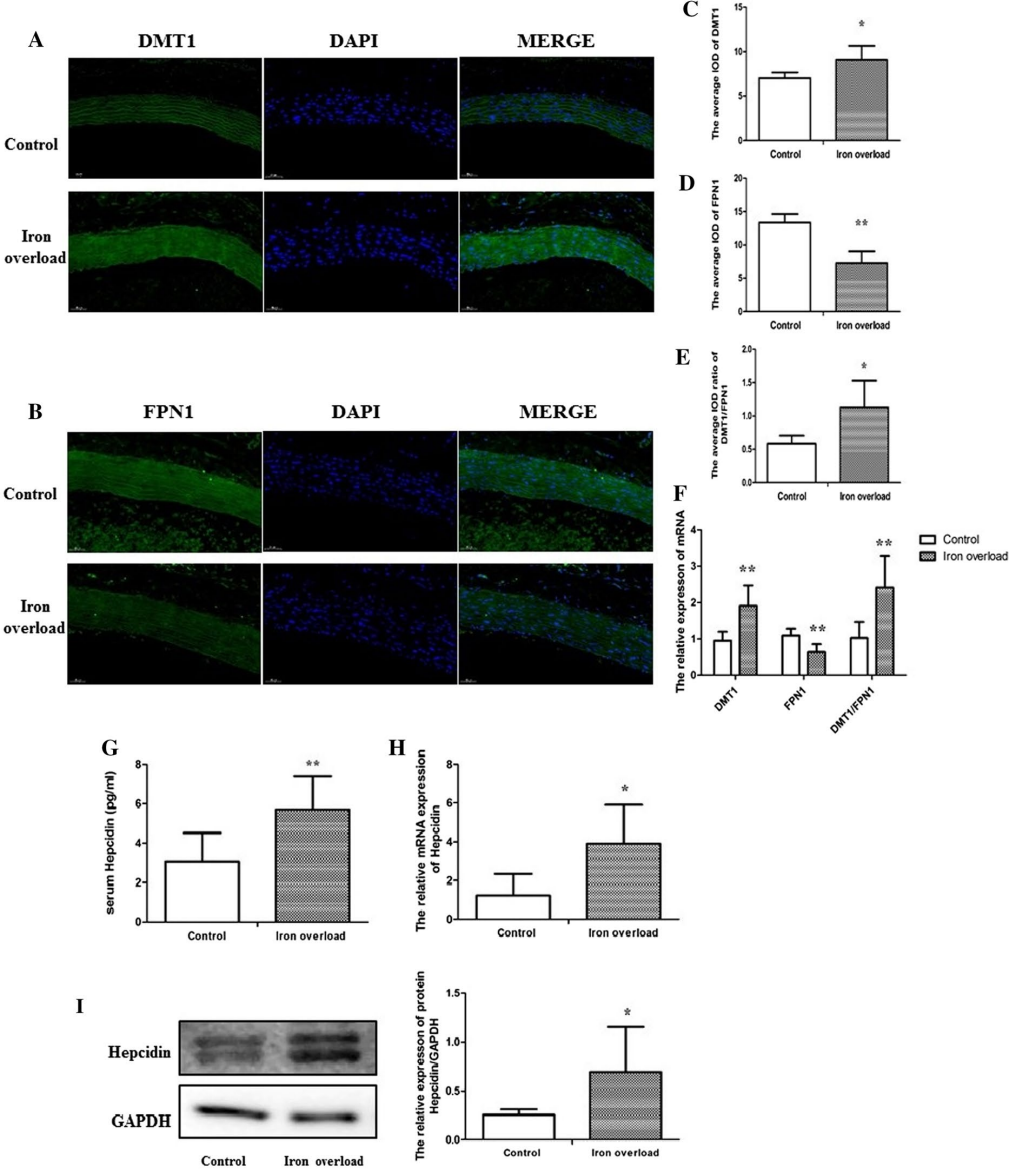
Expression of iron metabolism-related factors in aortic tissue of iron overload rats. **A** Immunofluorescence staining of DMT1 (200 × magnification), **B** Immunofluorescence staining of FPN1 (200 × magnification), **C** The average IOD of DMT1, **D** The average IOD of FPN1, **E** The average IOD ratio of DMT1 and FPN1, **F** The relative mRNA expression of DMT1, FPN1 and the ratio of DMT1 and FPN1, **G** Serum Hepcidin levels, **H** The relative mRNA expression of Hepcidin, **I** Western blot assay of Hepcidin. Data are expressed as means ± SD; n = 5–6 rats per group. *P < 0.05; **P < 0.01 *vs.* the control group

Furthermore, to examine the expression of iron metabolism-related factors, the immunofluorescence assay and qRT-PCR were conducted in aortic tissue of rat models of iron overload. In the iron overload group, the average IOD of divalent metal transporter 1 (DMT1) was higher (*P* < 0.05, Fig. 2A, C), while the average IOD of ferroportin1 (FPN1) was significantly lower than in the control group (*P* < 0.01, Fig. 2B, D). The average IOD ratios of DMT1 and FPN1 were also higher in the iron overload group (*P* < 0.05, Fig. 2E). Meanwhile, in the iron overload group, DMT1 mRNA expression and the relative mRNA expression ratio of DMT1 and FPN1 were significantly higher, and FPN1 mRNA expression was significantly downregulated than in the control group (*P* < 0.01, Fig. 2F).

In addition, the serum hepcidin level was increased in iron overload rats (*P* < 0.01, Fig. 2G). Compared with the control group, the relative mRNA (Fig. 2H) and protein (Fig. 2I) expression of hepcidin in aortic tissue were also higher (*P* < 0.05).

### Iron overload induced calcium depositions in rat aortic tissue

**Fig. 3:**
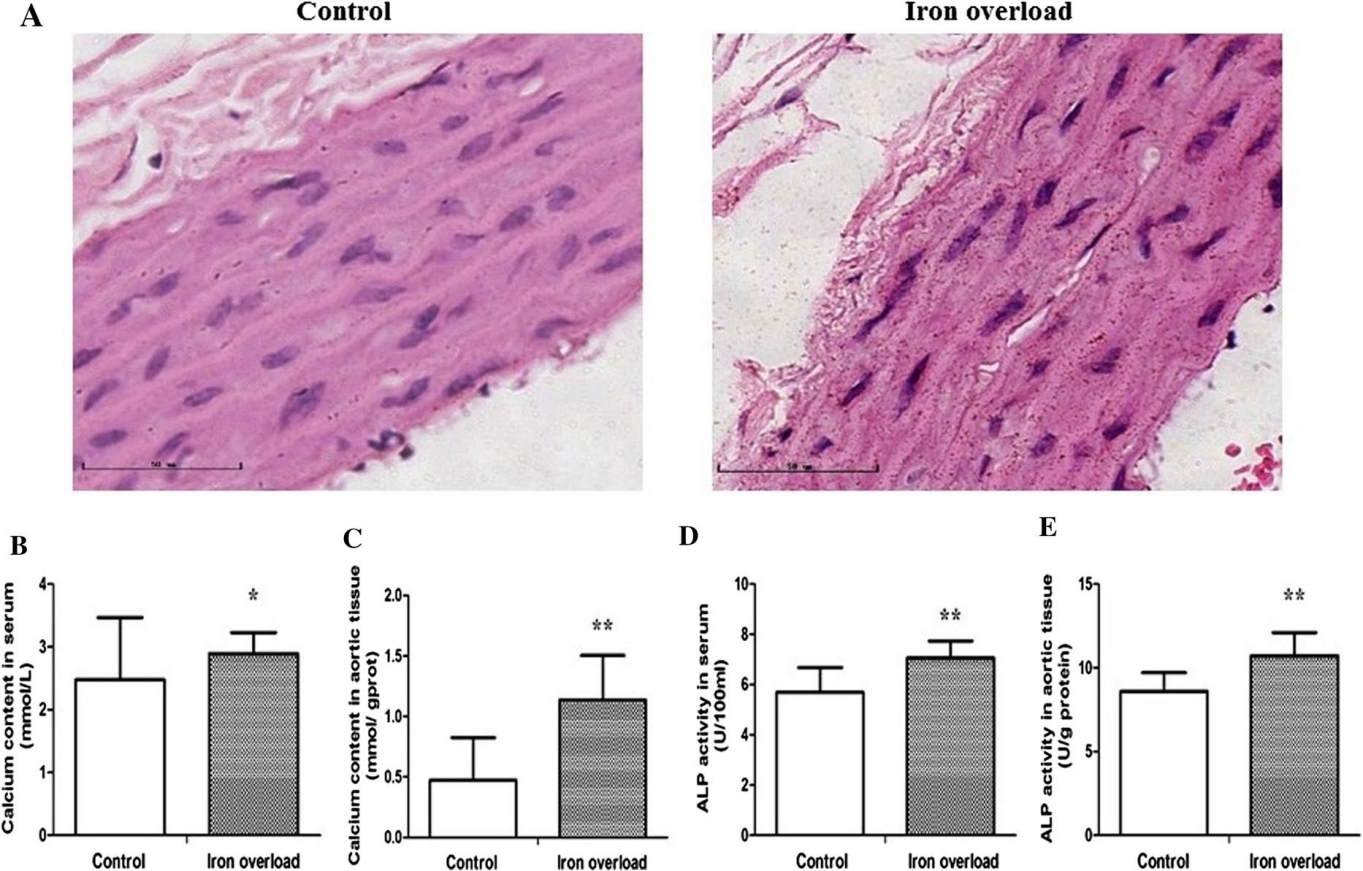
Iron overload induced calcium depositions in rat aortic tissue. **A** Von Kossa staining of rats’ aortic tissue (400 × magnification), **B** Calcium content in serum, **C** Calcium content in aortic tissue, **D** ALP activity in serum, **E** ALP activity in aortic tissue. Data are expressed as means ± SD; n = 6–7 rats per group. *P < 0.05; **P < 0.01 *vs.* the control group

To investigate the relationship between iron overload and calcification, we conducted Von Kossa staining and measured rat aortic tissue calcium and ALP activity levels. Von Kossa staining showed small brown calcium deposits in the rat aortic tissue (Fig. 3A). We also examined the calcium content in serum and aortic tissue. We found that the calcium content in serum (2.890 ± 0.337 mmol/L) (*P* < 0.05, Fig. 3B) and aortic tissue (1.136 ± 0.368 mmol/g protein) (*P* < 0.01, Fig. 3C) were both higher in the iron overload group than in the control group (2.474 ± 0.994, 0.472 ± 0.352). In addition, compared with the control group, higher ALP activity was observed both in serum and aortic tissue in the iron overload group (*P* < 0.01, Fig. 3D, E).

### Correlations between calcium content and iron levels and iron metabolism-related factors gene expression in aortic tissue

**Fig. 4:**
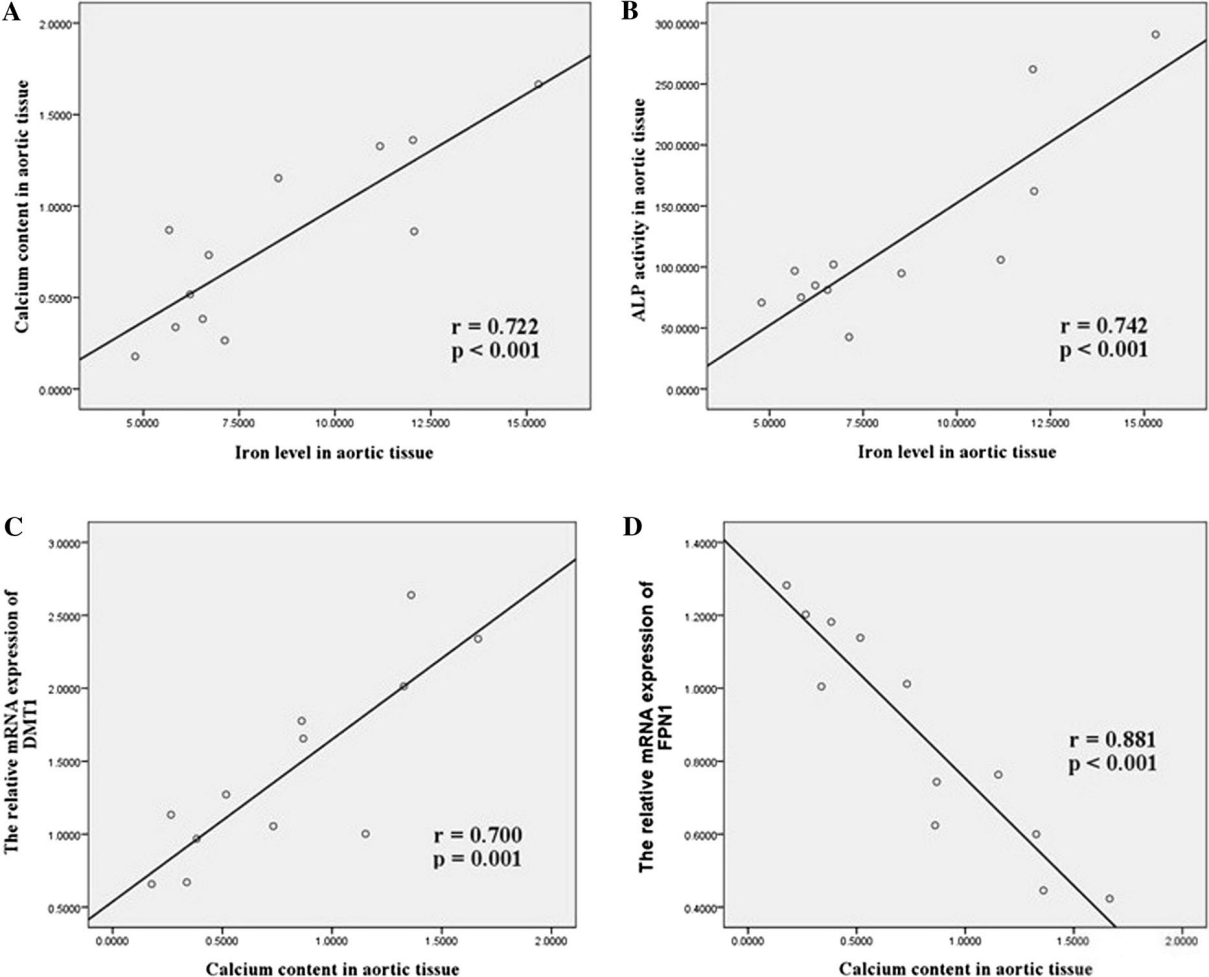
Correlations between calcium content and iron levels and iron metabolism-related factors gene expression in aortic tissue. *DMT1* Divalent metal transporter 1, *FPN1* Ferroportin1

We further assessed the relationships between calcium content and iron levels and iron metabolism-related factors gene expression in aortic tissue. As shown in Fig. 4, iron level highly correlated with calcium content (*r* = 0.722, *P* < 0.001) and ALP activity (*r* = 0.742, *P* < 0.001) in aortic tissue. Calcium content was significantly correlated with DMT1 gene expression (*r* = 0.700, *P* = 0.001), but inversely correlated with FPN1 gene expression (*r* = 0.881, *P* < 0.001) in aortic tissue.

### Iron overload inhibits the expression of α-SMA in rat aortic tissue

**Fig. 5:**
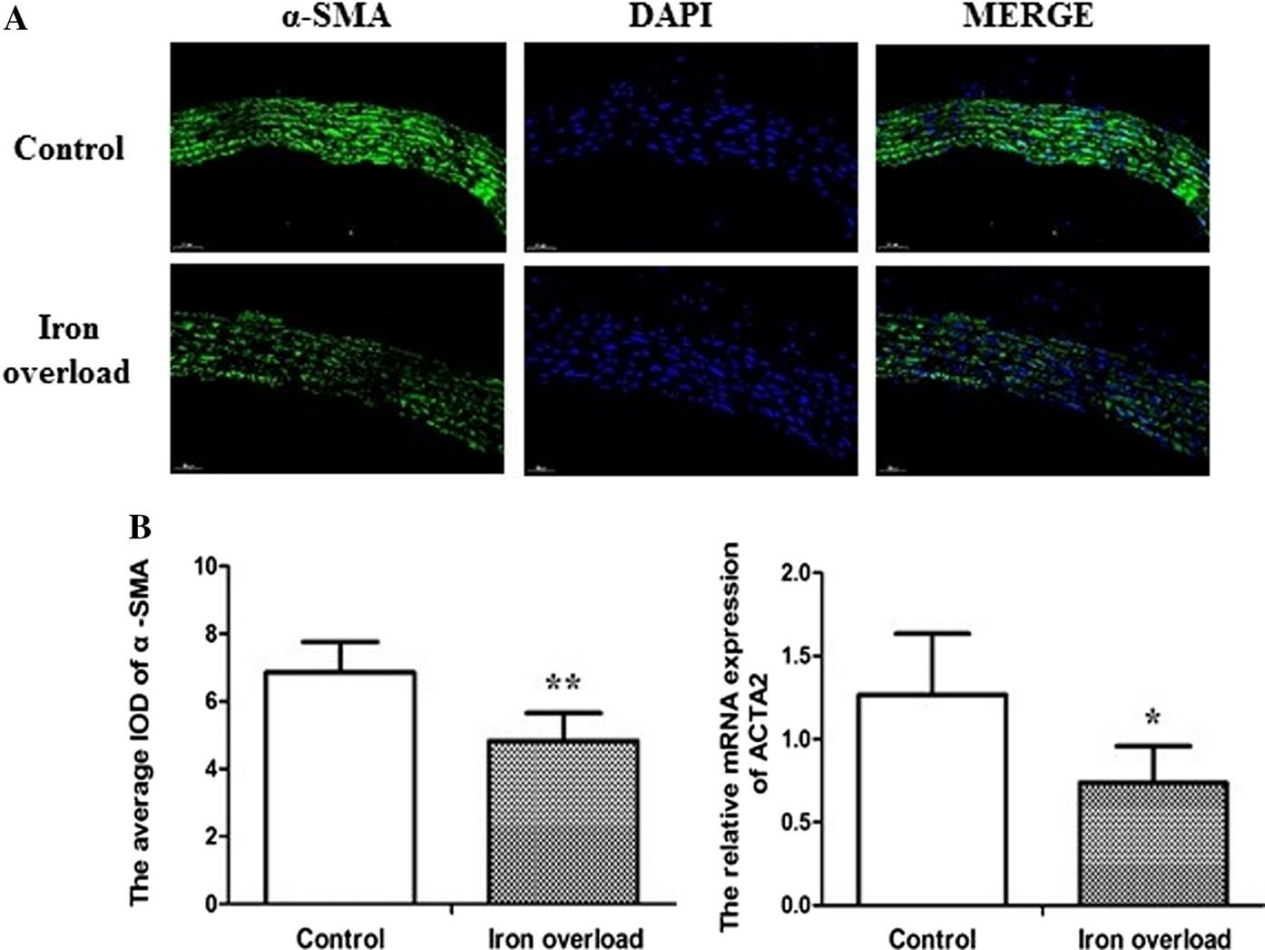
Iron overload inhibits the expression of α-SMA in rat aortic tissue. **A** Immunofluorescence staining of α-SMA (200 × magnification), **B** The average IOD of α-SMA, **C** The relative mRNA expression of α-SMA. Data are expressed as means ± SD; n = 6–7 rats per group. *P < 0.05; **P < 0.01 *vs.* the control group

Given the established relationship between VC and the osteogenic transformation of VSMCs, we quantified the expression of alpha-smooth muscle actin (α-SMA) in rat aortic tissue. As shown in Fig. 5, compared with the control group, both the average IOD (*P* < 0.01) and the relative mRNA expression of α-SMA (*P* < 0.05) were decreased in the iron overload group.

### Expression of factors related to calcification in aortic tissue of iron overload rats

**Fig. 6:**
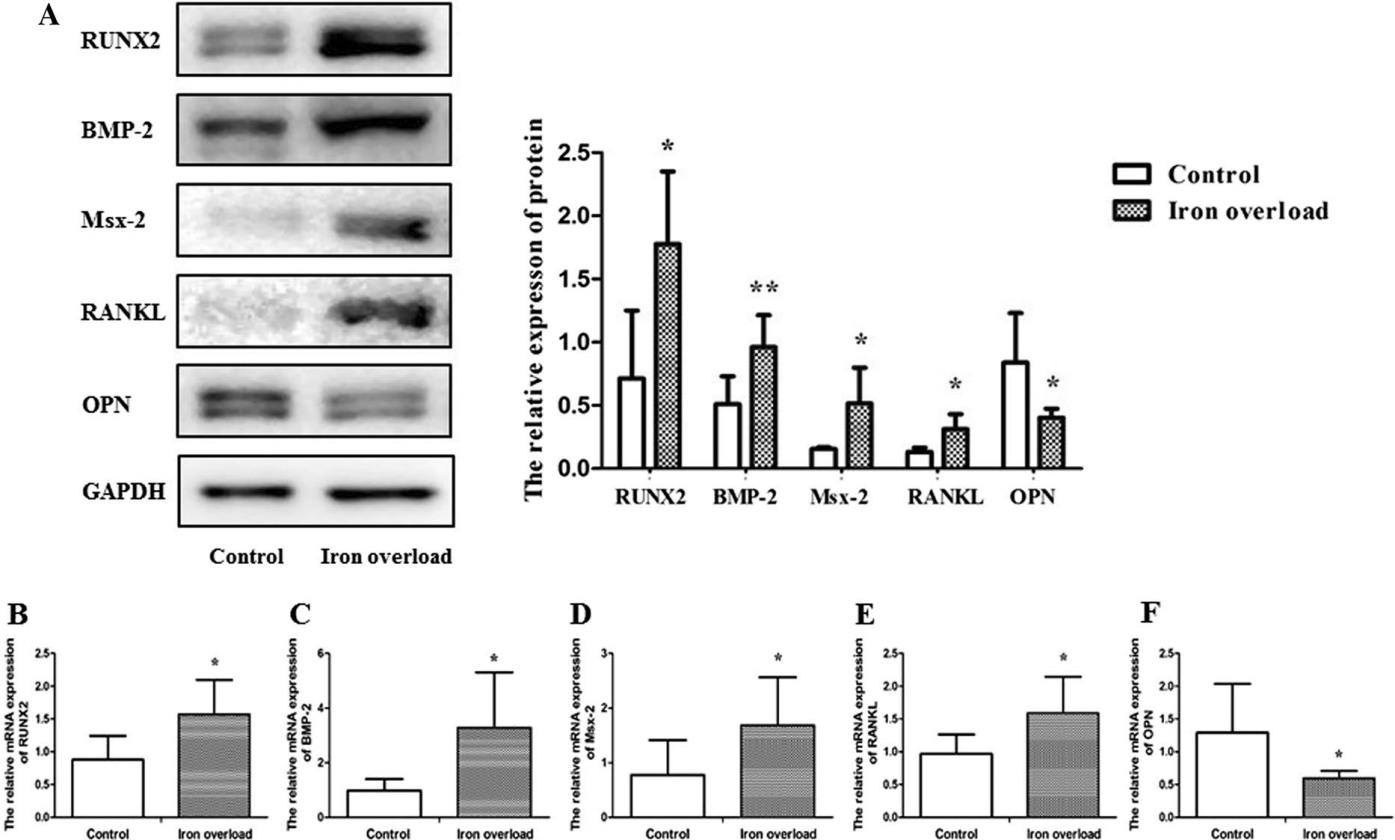
Expression of factors related to calcification in aortic tissue of iron overload rats. **A** Western blot assay of RUNX2, BMP-2, Msx-2, RANKL, and OPN. The relative mRNA expression of RUNX2 **B**, BMP-2 **C**, Msx-2 **D**, RANKL **E**, and OPN **F**. Data are expressed as means ± SD; n = 5–6 rats per group. *P < 0.05; **P < 0.01 *vs.* the control group

Based on the above findings, we further assessed the expression of osteogenic marker proteins, including runt-related transcription factor 2 (RUNX2), bone morphogenetic protein 2 (BMP-2), Msx-2, nuclear factor-kappa B ligand (RANKL) and calcification inhibitor osteopontin (OPN). Compared with the control group, iron overload increased the protein expression of RUNX2 (*P* < 0.05), BMP-2 (*P* < 0.01), Msx-2 (*P* < 0.05), RANKL (*P* < *0.05*) and decreased the OPN protein expression (*P* < 0.05) (Fig. 6A). The mRNA expression of factors related to calcification yielded consistent results with the western blot assay (*P* < 0.05*,* Fig. 6B–F).

### Iron overload increases the expression of IL-24 in rat aortic tissue

**Fig. 7:**
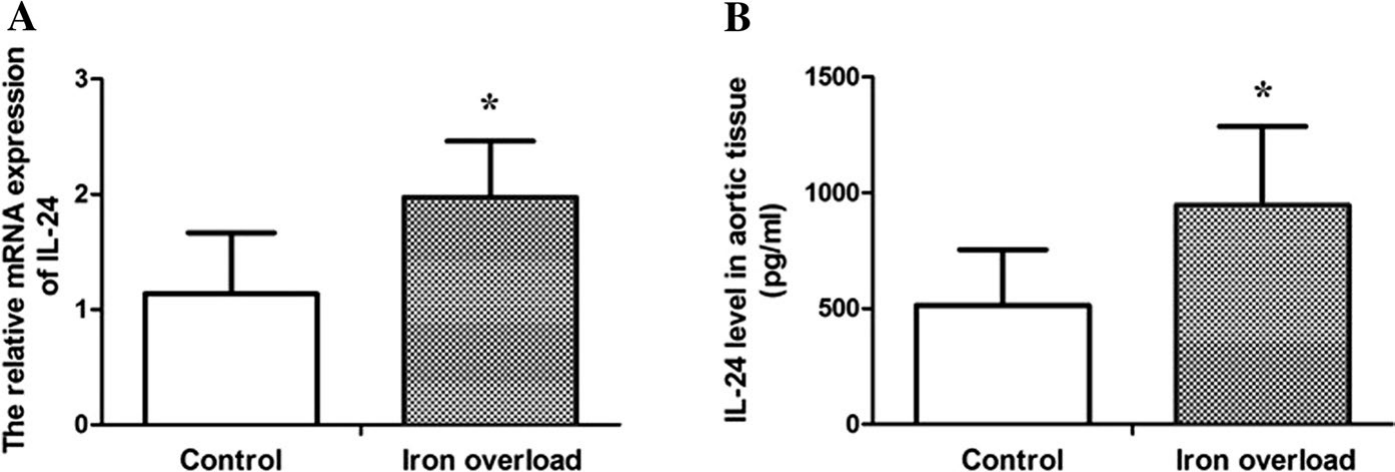
Iron overload increases the expression of IL-24 in rat aortic tissue. **A** The relative mRNA expression of IL-24, **B** IL-24 protein levels in aortic tissue. Data are expressed as means ± SD; n = 5–6 rats per group. *P < 0.05 vs. the control group

The expression of IL-24 in rat aortic tissue were evaluated by real-time PCR and Elisa. As shown in Fig. 7, the IL-24 gene expression was increased by iron overload (*P* < 0.05) (Fig. 7A). The IL-24 protein level was also increased in aortic tissue of iron overload group which was well consistent with the result of IL-24 gene expression (*P* < 0.05) (Fig. 7B).

## Discussion

It is well-recognized that iron metabolism plays an important role in maintaining the balance between iron uptake, transport, storage and utilization in our body (Ghio et al. 2021). Current evidence suggests that iron homeostasis is a highly regulated process essential to human health (Bogdan et al. 2016). Excessive iron has been recognized as a risk factor for tissue damage (Talvio et al. 2021). It has been shown that TSAT is a more sensitive indicator for diagnosing iron overload (Shander et al. 2012); When the TSAT exceeds 80–85%, non-transferrin-bound iron appears in the serum and can cause organ damage due to its high toxicity (Hiroshi 2018). In the present study, we investigated iron deposition and iron levels in aortic tissue of iron overload rat models. As expected, significant iron deposition was found in the aortic tissue. Moreover, we found that the serum levels of iron, ferritin and TSAT were higher in the iron overload group than in the control group, suggesting excessive iron accumulation in the body and the aortic tissue of iron overload rat models. The serum levels of BUN and CRE as the biomarkers of renal function were elevated in iron overload rats. Iron overload also increased the serum levels of ALT and AST as the biomarkers of hepatic function and decreased the serum Alb levels. These results suggested that both kidney and liver were damaged by excessive iron. In addition, iron overload induced low I-PTH secretion and high level of iP in serum. The results of iron overload on serum biochemistry parameters were inconstant. Kudo et al. demonstrated that iron overload lowered the liver and the renal function with low level of iP (Kudo et al. 2008). Matsushima et al. described that iron overload induced low serum levels of PTH and iP (Matsushima et al. 2003). However, after 5.0% of iron lactate in food intake, it showed no hepatic damage and renal dysfunction, but the serum iP level was increased (Fujimori et al. 2004). Therefore, in our study, we believe that iron overload, especially when the TSAT was nearly 80%, influenced the hepatic and renal metabolism, and really induced hepatic and renal damage. Renal disfunction can induced the high serum levels of calcium and phosphorus. High extracellular calcium concentration suppresses PTH secretion (Chang et al. 1997). And low PTH promotes the reabsorption of phosphorus from the urine. In a word, the above-mentioned reasons potentially lead to the hyperphosphatemia. But the relationship and mechanism of serum biochemistry parameters remains to be further studied.

DMT1 was the first mammalian transmembrane iron transporter identified in 1997 (Andrews 1999), widely distributed in the duodenum, liver and erythroid cells, and in major organs such as the kidney, lung, heart, brain, playing an important role in iron uptake in these tissues (Mims and Prchal 2005). FPN1 is a multiple-transmembrane domain protein first documented as a new cellular iron export protein in mammals in 2000 (Donovan et al. 2000). FPN1 is reportedly expressed in all cells that export iron, including duodenal enterocytes, white blood cells, Kupffer cells, brain astrocytes and placental cells, and is involved in erythrophagocytosis (Rice et al. 2009). Importantly, DMT1 and FPN1 have been validated to regulate iron homeostasis in tissues (Leong et al. 2003). Although the role of DMT1 and FPN1 has been established in duodenum tissue, their role in other tissues remains understudied. Gisela et al. (Giorgi et al. 2015) demonstrated that FPN1 expression was increased and DMT1 levels were unaltered in mice lung during exogenous iron overload. Li et al. 2018 examined the localization of DMT1 and FPN1 in bone tissue of rats found that DMT1 and FPN1 expression was significantly downregulated during exogenous iron overload. In contrast, it has been shown that cardiac expression of DMT1 increased and FPN1 decreased during exogenous iron overload, respectively (Zhang et al. 2021). In our study, we found that DMT1 and FPN1 were widely distributed in the aortic tissue of rats. In addition, exogenous iron overload upregulated the expression of DMT1 and downregulated the expression of FPN1 in rat aortic tissue. Overall, our results provide compelling evidence that iron accumulates in the aortic tissue of rats during iron overload. Further studies are warranted to explore the expression changes of DMT1 and FPN1 in different tissues during iron overload.

In recent years, hepcidin, as an important regulator of iron homeostasis, has been getting much attention in iron metabolism studies. By targeting FPN1 which is the only-known exporter of iron at the surface of cells, hepcidin downregulated the expression of FPN1, reduced the export of iron from cells to circulation, and thereby regulates the concentration of iron in the circulation (Wunderer et al. 2020). Hepcidin is upregulated by iron overload and inflammation (Prentice 2017). In clinical studies, the increased hepcidin level was significantly associated with arterial stiffness and cardiovascular events (Afsar et al. 2021). In our study, we detected the expression of hepcidin in serum and the aortic tissues. The results demonstrated that iron overload induced high levels of hepcidin both in serum and aortic tissues, with the degradation of FPN1.

In the present study, calcium depositions were found in the aortic tissue of iron overload rats with high aortic tissue and serum calcium levels. ALP is a ubiquitous enzyme that catalyzes the hydrolysis of phosphomonoesters with the release of Pi and is critical for physiological and pathological biomineralization (Hunter et al. 2011). It is widely thought that ALP plays a key role in VC, given that elevated serum ALP activity has been associated with calcific arteriolopathy (Lomashvili et al. 2004). In the present study, we assessed aortic tissue and serum ALP activity and substantiated that iron overload resulted in elevated ALP activity in the aortic tissue and serum of rats.

We further assessed the correlation between iron and calcium in aortic tissue. Our results showed iron levels were tightly correlated with calcium content and ALP activity in aortic tissue, and there were also strong correlations between calcium content and iron metabolism-related factors. Maya et al. (OttoDuessel et al. 2011) reported cardiac iron uptake was strongly correlated with cardiac calcium stores in a murine model of iron overload, suggesting that cardiac calcium and iron are related. Therefore, our study confirms that iron and calcium have strong relationship in cardiovascular system, and excess iron may play a role in the process of VC.

VSMCs are well-established to have different subtypes that express contractile proteins such as α-SMA, SM-22a, SM myosin heavy chains SM-1and SM-2, calponin, and smoothelin. There is ample evidence corroborating that the phenotypic transition of VSMCs, from a contractile phenotype into an osteochondrogenic phenotype, plays an important role in VC (Leopold 2015; Sun et al. 2020). The change from the contractile phenotype to osteochondrogenic phenotype is characterized by loss of contractile markers (α-SMA, etc.) and gain of osteochondrogenic markers (Durham et al. 2018). BMP-2 belongs to the transforming growth factor-beta subfamily, and it has been demonstrated that BMP-2 is a crucial cytokine that regulates osteoblast differentiation (Matsubara et al. 2008). Importantly, RUNX2 belongs to the runt-related transcription factor family and is essential for osteoblast differentiation (Sun et al. 2012; Lin et al. 2015). BMP-2 is known to control the expression of RUNX2 and a homeobox gene, Msx-2 (Matsubara et al. 2008; Shao et al. 1117). Osteoprotegerin (OPG)/receptor activator of NF-κB ligand (RANKL) system is another key regulation system that controls the bone formation and are also involved in the arterial calcification. Studies have shown that RANKL increased BMP-2 release to promote osteoblastic activity in human VSMCs (Davenport et al. 2016). Several studies reported increased BMP-2, RUNX2, Msx-2 and RANKL expression in VC, suggesting that they play an important role in VC (Liou et al. 2020; Zhou et al. 2019; Shao et al. 2005; Hortells et al. 2017). Accordingly, we further assessed the expression of BMP-2, RUNX2, Msx-2 and RANKL in the aortic tissues of iron overload rat models. We found that iron overload could increase the mRNA and protein expression of BMP-2, RUNX2, Msx-2 and RANKL. Meanwhile We also detected the expression of OPN, which is one of the important negative regulators of calcification (Scatena et al. 2007). Our results showed iron overload could decrease the mRNA and protein expression of OPN. However, the effect of exogenous iron overload on the osteogenic differentiation of calcification in vitro studies showed conflict results. Balogh et al. (Balogh et al. 2016) demonstrated that iron overload attenuated Pi-mediated calcification and decreased the expressions of RUNX2 and OCN in human bone marrow mesenchymal stem cells. And iron citrate can reduced high Pi-induced Ca deposition in rat VSMCs (Ciceri et al. 2016). But Kawada et al. reported that iron overload induced calcification and elevated the expression of BMP-2 in human aortic VSMCs with the effect of Pi, and IL-24 was increased during the calcification process induce by excessive iron (Kawada et al. 2018). Our results are consistent with Kawada’s in vitro results. Our findings suggest that iron overload induces renal function damage, leads to hypercalcemia and hyperphosphatemia, and then promotes the progressive VC by inducing osteoblast differentiation factors and downregulating the inhibitory factor of calcification. Increased IL-24 may play a role in the process of iron-promoted calcification.

Nevertheless, this study has some limitations. Our study indicates that iron overload induced VC in aorta, but not involve whether deferoxamine attenuate iron overload-induced VC. We also have not considered the time course of the experiments. Further studies are required to clarify these problems.

## Conclusion

In conclusion, the present study demonstrated excessive iron accumulated in the aortic tissue and induced organs damage in iron overload rat models. The iron metabolism-related factors, DMT1, FPN1, and hepcidin were widely distributed in the rat aortic tissues and were significantly dysregulated during iron overload. Importantly, iron overload impairs renal function, induces hypercalcemia and hyperphosphatemia, and then leads to calcium deposition in aortic tissues and may promote the progression of VC by inducing osteoblast differentiation factors and downregulating the inhibitory factor of calcification. Meanwhile increased IL-24 may play a role in the process of iron-promoted calcification. These findings highlight the key role of excessive iron in the pathological process of VC and provide a potential idea for the treatment of VC. Nonetheless, the role of iron in the development of VC is largely unclear, emphasizing the need for further studies to explore the mechanisms of VC.
